# VITA-D: Cholecalciferol substitution in vitamin D deficient kidney transplant recipients: A randomized, placebo-controlled study to evaluate the post-transplant outcome

**DOI:** 10.1186/1745-6215-10-36

**Published:** 2009-05-29

**Authors:** Ursula Thiem, Georg Heinze, Rudolf Segel, Thomas Perkmann, Franz Kainberger, Ferdinand Mühlbacher, Walter Hörl, Kyra Borchhardt

**Affiliations:** 1 Department of Internal Medicine III, Division of Nephrology and Dialysis, Medical University of Vienna, Währinger Gürtel 18-20, 1090 Vienna, Austria; 2 Core Unit for Medical Statistics and Informatics, Section of Clinical Biometrics, Medical University of Vienna, Währinger Gürtel 18-20, 1090 Vienna, Austria; 3 Hospital Pharmacy of the Vienna General Hospital, Währinger Gürtel 18-20, 1090 Vienna, Austria; 4 Department of Laboratory Medicine, Medical University of Vienna, Währinger Gürtel 18-20, 1090 Vienna, Austria; 5 Department of Diagnostic Radiology, Medical University of Vienna, Währinger Gürtel 18-20, 1090 Vienna, Austria; 6 Department of Surgery, Division of Transplantation, Medical University of Vienna, Währinger Gürtel 18-20, 1090 Vienna, Austria

## Abstract

**Background:**

Vitamin D does not only regulate calcium homeostasis but also plays an important role as an immune modulator. It influences the immune system through the induction of immune shifts and regulatory cells resulting in immunologic tolerance. As such, vitamin D is thought to exert beneficial effects within the transplant setting, especially in kidney transplant recipients, considering the high prevalence of vitamin D deficiency in kidney transplant recipients.

**Methods/Design:**

The VITA-D study, a randomized, placebo-controlled, double-blind study with two parallel groups including a total of 200 kidney transplant recipients, is designed to investigate the immunomodulatory and renoprotective effects of cholecalciferol (vitamin D_3_) within the transplant setting. Kidney transplant recipients found to have vitamin D deficiency defined as 25-hydroxyvitamin D_3 _< 50 nmol per liter will be randomly assigned to receive either oral cholecalciferol therapy or placebo and will be followed for one year. Cholecalciferol will be administered at a dose of 6800 International Units daily over a time period of one year.

The objective is to evaluate the influence of vitamin D_3 _substitution in vitamin D deficient kidney transplant recipients on the post-transplant outcome. As a primary endpoint glomerular filtration rate calculated with the MDRD formula (modification of diet in renal disease) one year after kidney transplantation will be evaluated. Incidence of acute rejection episodes, and the number and severity of infections (analyzed by means of C-reactive protein) within the first year after transplantation will be monitored as well. As a secondary endpoint the influence of vitamin D_3 _on bone mineral density within the first year post-transplant will be assessed. Three DXA analyses will be performed, one within the first four weeks post-transplant, one five months and one twelve months after kidney transplantation.

**Trial Registration:**

ClinicalTrials.gov NCT00752401

## Background

### Vitamin D and vitamin D deficiency

Vitamin D is a steroid hormone important for the regulation of calcium homeostasis and bone metabolism. There are two ways of obtaining vitamin D, either from the diet or by cutaneous production. Exposure of the skin to ultraviolet light induces the conversion of 7-dehydrocholesterol to previtamin D_3 _which then spontaneously isomerizes to vitamin D_3 _(cholecalciferol). Two hydroxylation steps in the metabolic activation of vitamin D_3 _are necessary to convert it into its physiologically active form 1,25-dihydroxyvitamin D_3 _[calcitriol; 1,25(OH)_2_D_3_]. Vitamin D_3 _is first hydroxylated in the liver to 25-hydroxyvitamin D_3 _[calcidiol, 25(OH)D_3_] and subsequently converted to 1,25-dihydroxyvitamin D_3 _by 1α-hydroxylase in the kidney [1].

25-hydroxyvitamin D_3 _is considered the most reliable indicator of vitamin D status as it reflects an individual's dietary intake of vitamin D on the one hand and the endogenous production in the skin on the other hand [2]. However, endogenous synthesis of vitamin D_3 _is more important to achieve adequate body stores than dietary intake due to the fact that vitamin D is found in a limited variety of foods, mainly oily fish. Casual exposure to sunlight supplies about 90% of the vitamin D requirement for most people [3].

Especially, the elderly and chronically ill are at high risk to develop vitamin D deficiency due to poor dietary vitamin D intake on the one hand and their often modest outdoor activities and the lack of exposure to sunlight on the other hand [4,5]. Moreover, the capacity of human skin to produce vitamin D_3 _decreases in elderly subjects compared to younger adults [3]. In a hospital setting, Thomas et al. reported 57% of hospitalized patients to be vitamin D deficient defined as 25-hydroxyvitamin D_3 _≤ 37 nmol per liter [4].

Accordingly, a high prevalence of vitamin D deficiency was found in predialysis patients with moderate and severe chronic kidney disease as well as in patients undergoing hemodialysis [6,7]. Analyses of pretransplant 25-hydroxyvitamin D_3 _levels showed that only about 15% of kidney transplant recipients have adequate vitamin D levels at the time of transplantation [8,9]. After kidney transplantation vitamin D deficiency remains a common problem as kidney transplant recipients are advised to avoid direct sun exposure because of the high risk of skin cancer due to immunosuppressive therapy [10]. Considering the fact that also dietary calcium and vitamin D intakes are often inadequate in kidney transplant recipients [11] they are at especially high risk to develop vitamin D deficiency. Querings et al. analyzed serum 25-hydroxyvitamin D_3 _levels in 31 kidney transplant recipients who all protected themselves from sun exposure compared with an age- and gender-matched control group at the end of winter. Serum 25-hydroxyvitamin D_3 _levels were significantly lower in kidney transplant recipients compared with controls, with 10 out of 31 having undetectable serum 25-hydroxyvitamin D_3 _levels [12]. For long term kidney transplant recipients Stavroulopoulos et al. reported vitamin D insufficiency (40–75 nmol per liter) to be present in 43%, deficiency (12–39 nmol per liter) in 46% and severe deficiency (< 12 nmol per liter) in 5% [13].

### Substitution of vitamin D deficiency

Considering the high prevalence of vitamin D deficiency in kidney transplant recipients the investigation of vitamin D substitution is of particular interest and relevance to renal transplant patients.

Chronic kidney disease is accompanied by decreased production of the active form of vitamin D, 1,25-dihydroxyvitamin D_3_, due to a reduction in renal parenchyma leading to decreased 1α-hydroxylase available for converting 25-hydroxyvitamin D_3_. Therefore, active vitamin D supplements have effectively been used in order to maintain calcium homeostasis and to decrease the risk of hyperparathyroidism in patients with chronic kidney disease.

However, it is still important to correct calcidiol deficiency as 25-hydroxyvitamin D_3 _serves as a substrate for 1α-hydroxylase not only in the kidney but also in several extra renal tissues. For instance, there is evidence that immune cells express 1α-hydroxylase and are therefore able to synthesize active vitamin D locally. Thus, these tissues are dependent on adequate 25-hydroxyvitamin D_3 _substrate for adequate local 1,25-dihydroxyvitamin D_3 _production [14].

As such, vitamin D is thought to be a potent immune modulator.

### Immunomodulatory properties of vitamin D

Besides its classical actions in calcium homeostasis and bone metabolism vitamin D plays an important role in the immune system [15,16]. Due to its potential to modulate the immune response vitamin D is thought to have beneficial effects within the transplant setting. Calcitriol was shown to improve graft function and graft survival in different models of transplantation and it is also assumed to lead to less acute rejection episodes [17]. Data relating infections to vitamin D status are sparse. There is some evidence that low 25-hydroxyvitamin D_3_levels are associated with an increased risk of infections [18,19].

Calcitriol acts as an inhibitor of the adaptive immune system with T-lymphocytes and antigen-presenting cells as key targets [20]. On the one hand 1,25-dihydroxyvitamin D_3 _is able to shift the T-cell response towards a T-helper 2 phenotype which is considered to be protective in transplantation [21] and on the other hand it induces dendritic cells with tolerogenic properties resulting in T-cell hyporesponsiveness. This means that dendritic cells modulated by 1,25-dihydroxyvitamin D_3 _are able to inhibit T-cell proliferation and their cytokine production (e.g. Interferon-γ), thus, inducing regulatory T-cells with suppressive activity rather than effector T-cells [22,23]. These effects are not limited to in vitro experiments, but could also be demonstrated in models of allograft rejection [24,25].

In clinical trials, a beneficial impact of calcitriol administration in kidney transplant recipients on graft function, overall graft survival and incidence of acute rejection episodes was demonstrated [26-29]. However, these studies are retrospective studies that do not provide definitive evidence of the assumed beneficial immunological effects. In a small prospective study Ardalan et al. investigated the effect of calcitriol started in the donor six days before donation and continued in recipient side for six months after transplantation. Expansion of CD4+ CD25+ regulatory T-cells was observed in the recipients [30] providing strong evidence for the immunomodulatory properties of active vitamin D in kidney transplant recipients.

### Renoprotective properties of vitamin D

In several studies beneficial effects of active vitamin D on progression of chronic kidney disease were documented, either by interfering with the transforming growth factor beta 1 (TGF-β1) signaling pathways or with the renin-angiotensin-aldosterone system, both considered to be important modulators of the progression of renal insufficiency [31,32].

1,25-dihydroxyvitamin D_3 _was shown to function as a negative regulator of the renin-angiotensin system in animals [33]. Recently, Freundlich et al. demonstrated that paricalcitol, a calcitriol analog, leads to a reduction in mRNA levels and protein expression of angiotensinogen, renin, and renin receptor in the 5/6 nephrectomized rat model of kidney disease resulting in less glomerular and tubulointerstitial damage, hypertension and proteinuria [34].

In diverse rat models of kidney disease 1,25-dihydroxyvitamin D_3 _was reported to attenuate the development of glomerulosclerosis and progression of albuminuria accompanied by a lower expression of TGF-β1 in the kidney [35,36].

Furthermore, the podocyte was identified as an important target for the action of 1,25-dihydroxyvitamin D_3_. Calcitriol reduces podocyte loss and changes of podocyte foot process structure leading to a decreased albumin excretion [37,38].

A recent clinical trial indicated that paricalcitol reduces proteinuria in patients with chronic kidney disease. Agarwal et al. analyzed the effect of paricalcitol on proteinuria in 195 chronic kidney disease patients stage 3 and 4 compared to placebo. After six months 51% of the paricalcitol treated subjects compared to 25% of the placebo treated subjects were found to have a reduction in proteinuria independent from the use of inhibitors of the renin-angiotensin-aldosterone system [39].

TGF-β1 is furthermore considered to play an important role in the development of chronic allograft nephropathy. Chronic allograft nephropathy is characterized by interstitial fibrosis and glomerulosclerosis and frequently leads to late allograft loss in kidney transplantation [40]. There is evidence that calcitriol interferes with TGF-β1 mediated gene and protein expression and therefore influences the effects of TGF-β1 in chronic allograft nephropathy [41]. Hullett et al. investigated the efficacy of active vitamin D as monotherapy to prolong allograft survival and preserve renal function in a rat model of chronic allograft nephropathy. 1,25-dihydroxyvitamin D_3 _not only was reported to prolong graft survival but also to prevent histological changes typically found in chronic allograft nephropathy by altering TGF-β1 and matrix regulating molecules [42]. In clinical studies, patients with chronic allograft nephropathy were significantly linked with persistently higher plasma TGF-β1 levels [43].

1,25-dihydroxyvitamin D_3 _obviously has an impact on innate and adaptive immunity, both responsible for acute rejection and chronic allograft nephropathy which is often observed to start only a few months after kidney transplantation.

## Methods

### Objectives

The proposed study is designed to evaluate whether cholecalciferol substitution in vitamin D deficient kidney transplant recipients has beneficial effects upon the post-transplant outcome compared to placebo. As a primary outcome we will evaluate the impact of cholecalciferol on

- graft function (analyzed using glomerular filtration rate calculated with the MDRD formula as well as albuminuria) one year after kidney transplantation

- the incidence of acute rejection episodes within the first year after kidney transplantation, and

- the frequency and severity (analyzed by means of C-reactive protein levels) of infections within the first year after kidney transplantation.

As a secondary objective we will assess whether cholecalciferol substitution influences the common decline of bone mineral density within the first year after kidney transplantation [44] analyzed by means of absolute bone mineral density (g/cm^2^).

Moreover, courses of calcium levels will be observed in order to assess the safety of cholecalciferol administration at 6800 International Units (IU).

### Study design and setting

The VITA-D study is a single-center, randomized, placebo-controlled parallel group study with blinding of patients, study staff, and outcome assessors. Patient recruitment, kidney transplants, postoperative care, and follow-up are conducted at the Medical University of Vienna. The study is planned to start in January 2009. There are about 150 kidney transplants performed annually at the Medical University of Vienna. Approximately 30% of all kidney transplant recipients are expected to be vitamin D deficient defined as 25-hydroxyvitamin D_3 _< 50 nmol per liter. In case there are less than 60 subjects included in the trial after one year it is intended to recruit two other transplant centers.

### Study population and sample size

**Figure 1:**
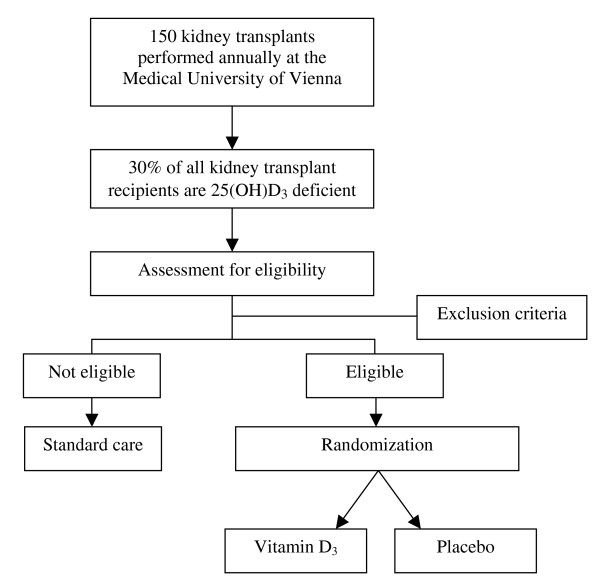
**Flowchart of the VITA-D study**.

The study sample will consist of 200 kidney transplant recipients found to be vitamin D deficient (25-hydroxyvitamin D_3 _< 50 nmol per liter) with 100 subjects in each study group. Inclusion and exclusion criteria are detailed in the Appendix. The recruitment phase will last approximately four years with a follow-up period of one year for each subject. A flowchart of the VITA-D study is shown in Figure 1. The sample size calculation was performed according to the following assumptions: Based on the Vienna General Hospital database we concluded that the mean serum creatinine level one year after transplantation is x_1 _= 1,9 mg per deciliter with sigma, the common standard deviation, estimated to be σ = 0.5. An expert panel evaluated that a difference in serum creatinine levels of 0,2 mg per deciliter would represent a significant clinical improvement for the patient. Assuming that α = 0.05 and the power = 0.80 a sample size of 99 for each sample was calculated.

### Randomization and blinding

If all inclusion and none of the exclusion criteria are met and informed consent has been obtained kidney transplant recipients will be included in the study and allocated a randomization number according to a randomization list. Thus, the participant will be randomly allocated to one of the two treatment arms. The random allocation sequence will be computer-generated using the Randomizer for Clinical Trials 1.7.0 developed at the Institute for Medical Informatics, Statistics and Documentation, Medical University of Graz, Austria. Block randomization will be used in order to prevent imbalanced treatment group sizes. We will use blocks of six to which the two treatment modalities will be randomly allocated in the ratio 1:1. To ensure homogenous treatment groups randomization is stratified by 25-hydroxyvitamin D_3 _levels, with one sub-stratum of kidney transplant recipients found to have 25-hydroxyvitamin D_3 _levels between 25 and 50 nmol per liter and another of kidney transplant recipients with 25-hydroxyvitamin D_3 _levels below 25 nmol per liter.

Participants, investigators, and outcome assessors will be blinded to the allocated treatment. Blinding will be ensured by the use of investigational products that are identical in packaging, labeling, appearance, smell, and taste. Unblinding will only occur in case of emergency and at the conclusion of the study. In most cases, however, discontinuing the treatment should be sufficient without the need for unblinding. Access to the randomization list is restricted to the pharmacist involved in the study and a research assistant.

### Study intervention

Kidney transplant recipients found to have 25-hydroxyvitamin D_3 _levels below 50 nmol per liter and who fulfill all of the inclusion and none of the exclusion criteria will be randomized to receive either oral cholecalciferol therapy (Oleovit^® ^D_3_-drops) or a placebo solution. Treatment starts on day five after kidney transplantation. Cholecalciferol will be administered at a dose of 6800 IU daily over a time period of one year.

The only adverse drug reaction caused by vitamin D_3 _that may occur is the rise in blood levels of calcium. In case of hypercalcemia the dose can be reduced immediately as serum calcium levels are monitored routinely at close intervals (daily within the postoperative hospitalization, i.e. between two and four weeks on average, thereafter weekly until the fourth month after transplantation, and subsequently every two to four weeks depending on post-transplant complications). At serum calcium levels > 2,65 mmol per liter vitamin D_3 _administration will be reduced to 3600 IU per day. If calcium levels persist above 2,85 mmol per liter over a period of four weeks vitamin D_3 _administration will be discontinued and restarted when serum calcium levels declined to ≤ 2,65 mmol per liter with only 3600 International Units per day. If calcium levels remain high this may be attributed to a persistent or transient hypercalcemia due to hyperparathyroidism as transient hypercalcemia is observed in 20–50% in kidney transplant recipients. Thus, the subject will be tested with ultrasound and ^99m^Tc-methoxyisobutyl isonitrile (MIBI) scan. However, these subjects will not be excluded from the study.

### Concomitant medication

Prior to kidney transplantation the administration of all active vitamin D supplements which are prescribed to almost all patients on dialysis will be discontinued.

Most kidney transplant recipients are administered a triple immunosuppressive therapy consisting of either mycophenolatmofetil or mycophenolat-natrium, tacrolimus, and prednisone. Steroid boli, monoclonal, and polyclonal antibodies are administered to treat acute rejection episodes.

### Study procedures

#### Day 0

Prior to transplantation patients will be recruited from the acute dialysis ward. There, and previous to kidney transplantation, blood samples will be taken of all kidney transplant candidates as a matter of routine, including a blood sample for 25-hydroxyvitamin D_3 _analyses.

#### Day 1–5

If a kidney transplant recipient is found to have 25-hydroxyvitamin D_3 _levels below 50 nmol per liter information about the VITA-D study, its purpose, putative benefits, and possible risks will be given to the patient and he/she will be invited to participate in the trial. All subjects will be informed that the participation in the trial is voluntary and that the subject may refuse to participate or withdraw from the trial at any time without giving reasons, without loss of benefits. Kidney transplant recipients who do not agree to participate in the study will receive standard care.

Demographic and baseline data will be collected of all participants within the first five days after transplantation (gender, age, body mass index, cause of end stage renal disease, mode of renal replacement therapy, duration on dialysis).

#### Day 5

Kidney transplant recipients who agreed to participate in the study will be randomized to receive either oral vitamin D_3 _therapy or placebo.

#### Treatment in hospital

Kidney transplant recipients are treated in hospital between two and four weeks on average. Within this time period blood samples are taken daily as a matter of routine, including analyses of serum creatinine, C-reactive protein levels and calcium levels.

The first DXA (dual energy X-ray absorptiometry) analysis will be performed within the first four weeks after transplantation depending on postoperative complications. However, we aim at performing it within the first two weeks.

#### Outpatient treatment

After discharge kidney transplant recipients are seen once a week within the first four months after transplantation and are thereafter scheduled for visits every two to four weeks within the first year after transplantation. Blood samples are taken at every visit at the nephrological outpatient department.

#### Week 16

25-hydroxyvitamin D_3 _levels will be analyzed in order to check the response to treatment. In addition, eating habits and lifestyle (sun exposure, outdoor activities, and clothing) will be evaluated by means of a questionnaire.

#### After 5 months

The second DXA analysis will be performed.

#### After 12 months

The third DXA analysis will be performed.

**Figure 2:**
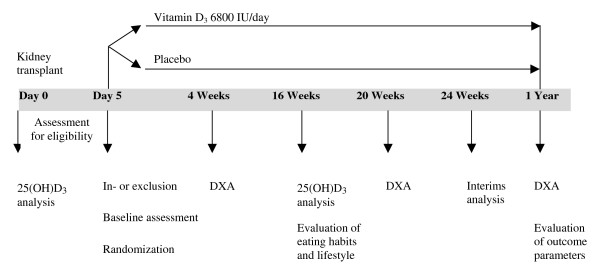
**Study procedures**.

A summary of all study procedures is shown in Figure 2.

### Statistical analysis

Baseline data will be expressed as mean values ± standard deviation for symmetrically distributed continuous variables, as median and 25th and 75th percentiles for asymmetrically distributed continuous variables, and as absolute and relative frequency for categorical variables.

Serum creatinine values after one year (the primary endpoint) will be compared between the groups by analysis of covariance (ANCOVA), taking into account as covariates creatinine value at randomization, 25-hydroxyvitamin D_3 _at baseline, and variables which, despite randomization, exhibit large group differences at baseline. ANCOVA will also be applied to the area under the curve of repeated measurements on serum creatinine, CRP, and bone mineral density during the first year of follow-up, using the same set of covariates. Categorical variables (number of acute rejection episodes and infections within the first year after transplantation) will be analyzed using χ^2^-test or fisher's exact test, as appropriate.

All analyses will be performed using an "efficacy subset analysis". Patients are included in this analysis if the study medication has been administered for more than seven days.

Moreover, courses of calcium levels will be described. Furthermore, the Bonferroni-Holm correction will be performed to preserve the global significance level of 5%.

Statistical analysis will be performed with SPSS.

### Interims analysis

An interims analysis will be performed after 6 months of follow-up for every subject. Within this analysis glomerular filtration rates between the two groups six months after transplantation and the incidence of acute rejection episodes within six months after transplantation will be analyzed. In case the interims analysis shows a significant difference between the two groups the study will be discontinued prematurely. The strict significance level of 0.001 for interim analysis requires no modification of the significance level for the main analysis [45]. The interims analysis will not break blinding.

### Approval of the ethics committee and the regulatory authority

The proposed study will be conducted in accordance with the Declaration of Helsinki, the Austrian law and subscribes to the principles outlined in the International Conference on Harmonization on Good Clinical Practice, 2002. Approvals were obtained by the Ethics Committee of the Medical University of Vienna and the Vienna General Hospital (Reference Number 213/2008) and the Austrian regulatory authority (Federal Office for Safety in Health Care, Austrian Agency for Health and Food Safety). Furthermore, the study has been registered in a public clinical trial database (ClinicalTrials.gov NCT00752401).

## Discussion

### Risk analysis

Considering current clinical trial data [46-48] the administration of vitamin D_3 _at a dose of 6800 IU per day is not expected to pose any risks of severe adverse health effects. Vitamin D intoxication is extremely rare and can only be caused by intake of excessively high doses. Doses of more than 50 000 IU per day can raise 25-hydroxyvitamin D_3 _levels to more than 370 nmol per liter and are associated with hypercalcemia and hyperphosphataemia [46]. However, a threshold for vitamin D toxicity has not been established yet. The tolerable upper intake level for vitamin D, which is the formal limit for safe intake of vitamin D and defined as "the amount of vitamin D that can be consumed by adults on a long-term basis with no anticipation of harm" [47] is set at 2000 IU per day. However, scientific evidence demonstrates that vitamin D is not toxic at intakes much higher than formerly considered unsafe. Based on clinical trial evidence that shows that prolonged intake of 10 000 IU per day of vitamin D_3 _is likely to pose no risk of adverse effects, the recommended upper intake level is set at 10 000 IU per day [48]. Moreover, there is no scientific evidence that the margin of safety for vitamin D_3 _is less for patients with renal disease than for the rest of the population [47].

## Conclusion

To date, clinical trials investigating vitamin D and its immunomodulatory properties within the transplant setting have been sparse, only a few small trials are available. We report the rationale, design, and methodology of the VITA-D study, the first prospective, randomized, placebo-controlled trial focusing on the immunomodulatory and renoprotective properties of vitamin D_3 _in kidney transplant recipients showing vitamin D deficiency. We will investigate whether or not vitamin D_3 _has beneficial effects upon graft function, the incidence of acute rejection episodes, and frequency and severity of post-transplant infections. If vitamin D_3 _substitution proves to have no relevant disadvantages and if effectiveness can be demonstrated then clinical practice and routine supplementation of vitamin D_3 _in vitamin D deficient kidney transplant recipients may be intensified.

## Competing interests

The study medication is provided by Fresenius Kabi Austria Gmbh, the marketing holder of cholecalciferol in Austria. However, the authors do not receive any reimbursement or financial benefits from the company and declare that they have no competing interests.

## Authors' contributions

UT made substantial contributions to the conception and design of the study and was essentially involved in drafting the study protocol and the manuscript.

GH participated in the design of the study by giving advice on statistics and will be involved in the statistical analyses.

RS will be in charge of the production, labeling and blinding of the investigational products.

TP participated in the design of the study by giving advice on laboratory procedures and will be involved in the laboratory analyses.

FK participated in the design of the study and helped to draft the manuscript.

FM participated in the design of the study and its coordination and helped to draft the manuscript.

WH participated in the design of the study and its coordination and helped to draft the manuscript.

KB is the principal investigator, responsible for recruitment and trial coordination. She developed the study idea and made substantial contributions to conception and design. Moreover, she was involved in drafting and revising the study protocol as well as this manuscript.

All authors will participate in the implementation or analysis of this study and approved the final manuscript.

## Appendix

### Eligibility criteria

#### Inclusion criteria

Age > 18

Deceased donor kidney transplant recipients

Only kidney transplant recipients

Vitamin D deficiency defined as 25-hydroxyvitamin D_3 _< 50 nmol per liter

#### Exclusion Criteria

Re-transplantation for the second time if the patient is highly immunized and therefore included in the aphaeresis program

Re-transplantation for the third or further time

Significant impaired intestinal resorption

History of inflammatory bowel disease: Crohn's disease, Ulcerative Colitis

Previous gastrectomy, small bowel or large bowel resection, intestinal bypass surgery

Severe liver disease: cirrhosis

HIV positive
